# Uniqueness of models from small-angle scattering data: the impact of a hydration shell and complementary NMR restraints

**DOI:** 10.1107/S1399004714013923

**Published:** 2015-01-01

**Authors:** Henry S. Kim, Frank Gabel

**Affiliations:** a Université Grenoble Alpes, IBS, 71 avenue des Martyrs, 38044 Grenoble, France; b CNRS, IBS, 71 avenue des Martyrs, 38044 Grenoble, France; c CEA, IBS, 71 avenue des Martyrs, 38044 Grenoble, France; d Institut Laue–Langevin, 38042 Grenoble CEDEX 9, France

**Keywords:** small-angle scattering, NMR restraints, hydration shell

## Abstract

Small-angle scattering (SAS) has witnessed a breathtaking renaissance and expansion over the past 15 years regarding the determination of biomacromolecular structures in solution. While important issues such as sample quality, good experimental practice and guidelines for data analysis, interpretation, presentation, publication and deposition are increasingly being recognized, crucial topics such as the uniqueness, precision and accuracy of the structural models obtained by SAS are still only poorly understood and addressed. The present article provides an overview of recent developments in these fields with a focus on the influence of complementary NMR restraints and of a hydration shell on the uniqueness of biomacromolecular models. As a first topic, the impact of incorporating NMR orientational restraints in addition to SAS distance restraints is discussed using a quantitative visual representation that illustrates how the possible conformational space of a two-body system is reduced as a function of the available data. As a second topic, the impact of a hydration shell on modelling parameters of a two-body system is illustrated, in particular on its inter-body distance. Finally, practical recommendations are provided to take both effects into account and promising future perspectives of SAS approaches are discussed.

## Abstract 2

The potentialities and limitations of biomacromolecular modelling of small-angle scattering data are reviewed and discussed with a focus on the impact of complementary NMR restraints and a hydration shell.

## 1. Introduction   

Small-angle scattering of X-rays (SAXS) or neutrons (SANS) can be used to obtain structural information on biomacromolecules at low resolution. In this technique, X-rays or neutrons are scattered from biological particles in solution. For isotropic systems (*i.e.* randomly orientated, non-interacting particles), the two-dimensional pattern measured on a detector can be azimuthally averaged (around the direct beam) and reduced into a one-dimensional curve *I*(*Q*) which encodes, after subtraction of the respective buffer curve, structural information on the particles in solution (Svergun *et al.*, 2013 ▶; Feigin & Svergun, 1987 ▶; Glatter & Kratky, 1982 ▶). *Q* = (4π/λ)sin(θ) is the momentum transfer of the radiation, where λ is the wavelength of the neutrons/X-rays and 2θ is the scattering angle.

**Figure 1:**
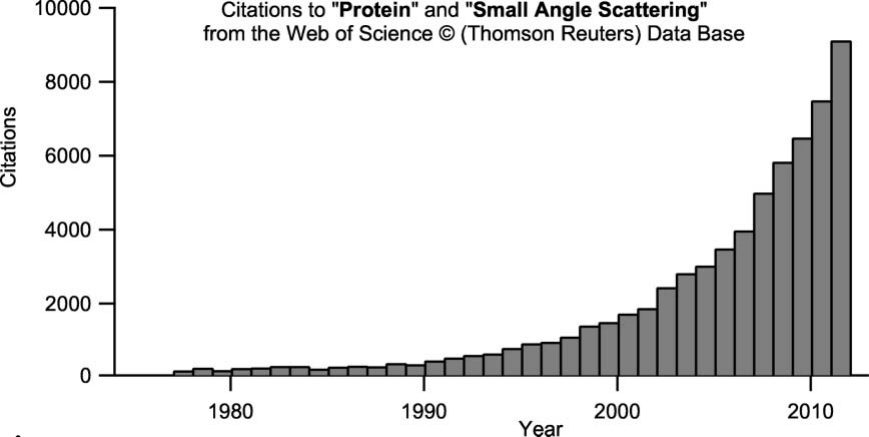
Citations of scientific publications using the search expression ‘protein’ + ‘small angle scattering’ (Web of Knowledge/Thomson Reuters).

Both SAXS and SANS have been applied to biomolecular solutions for several decades. The earliest biological work using SAXS included studies on the radius of gyration and shape of ovalbumin (Guinier, 1939 ▶). Biological SANS emerged in the 1960s/1970s with the advent of high-flux neutron sources (Stuhrmann, 1974 ▶; Engelman & Moore, 1972 ▶; Schneider *et al.*, 1969 ▶). A more detailed and complete overview of the pioneering experiments can be found elsewhere for SAXS (Kratky, 1963 ▶; Kratky & Pilz, 1972 ▶; Guinier, 1939 ▶) and SANS (Zaccai & Jacrot, 1983 ▶; Jacrot, 1976 ▶). Biological applications of SAS have witnessed an unprecedented expansion and renaissance since the 1990s (Fig. 1 ▶), mainly owing to advances in available sources, instruments and, importantly, in data analysis (Lipfert & Doniach, 2007 ▶; Koch *et al.*, 2003 ▶; Svergun & Koch, 2002 ▶). More recently, issues such as sample quality and good experimental practice (Jacques & Trewhella, 2010 ▶), as well as guidelines for data analysis, interpretation, presentation, publication and deposition, have received increasing attention (Trewhella *et al.*, 2013 ▶; Jacques, Guss & Trewhella, 2012 ▶; Jacques, Guss, Svergun *et al.*, 2012 ▶).

The present paper discusses, after a short introductory review, the equally important issues of the uniqueness of the structural models derived from SAS data, with a focus on NMR–SAS hybrid approaches and the influence of a hydration shell.

## 2. General considerations regarding the uniqueness of models derived from small-angle scattering data   

In structural biology, only two techniques can at present provide atomic resolution structures of biomacromolecules: X-ray (and neutron) crystallography and nuclear magnetic resonance (NMR). A third technique, electron microscopy (EM), is steadily approaching the threshold of atomic resolution, with the most recent single-particle cryo-EM structures reported close to 3 Å resolution (Milne *et al.*, 2013 ▶).

Amongst the complementary techniques that can provide structural information (at lower resolution) SAS has become increasingly popular, either being used alone or in combination with other techniques (Table 1 ▶). The fundamental theoretical reason why SAS in solution cannot provide atomic resolution information is a consequence of the averaging process of the scattered signal from arbitrarily oriented molecules in solution: the information content in real space is a pair distance distribution *p*(*r*) between scattering centres (electrons for X-rays and nuclei for neutrons), implying a loss of directionality with respect to crystallographic approaches (Guinier, 1994 ▶; Putnam *et al.*, 2007 ▶). Further practical reasons include the limited angular range of scattering data as well as their noise (Gabel, 2012 ▶). In this section, we briefly discuss and review the theoretical considerations regarding the limits of SAS by distinguishing two major cases: (i) *ab initio* determination of structures and (ii) rigid-body modelling of building blocks with known structure.

In general, the addition of structural restraints from other biophysical techniques (Zhao & Schuck, 2015 ▶) has been recognized to improve the quality and accuracy of structural models derived from SAS data (Perry & Tainer, 2013 ▶; Schneidman-Duhovny *et al.*, 2012 ▶; Petoukhov & Svergun, 2007 ▶) and has been applied to a multitude of systems over recent years (Table 1 ▶).

### 2.1. *Ab initio* shape determination   

After an early period (1930s–1960s) when SAXS data of biomacromolecules were interpreted in terms of simple geometric bodies (Kratky & Pilz, 1972 ▶), a first general shape-analysis approach was proposed in the early 1970s (Stuhrmann, 1970*a* ▶,*b* ▶) by approximating the particle surface using a truncated series of an appropriate set of orthonormal functions (spherical harmonics). The uniqueness of structural solutions can be discussed elegantly in terms of the mathematical properties of the generating functions (Stuhrmann, 1970*a* ▶,*b* ▶): the back-calculated scattered intensity is independent of a rotation of the *l*th partial structure. More generally, all symmetry operations of a particle structure that leave the *p*(*r*) function unchanged, including the special classes of mirror and point symmetry operations, yield equivalent SAS data owing to the equivalence of *I*(*Q*) and *p*(*r*) (Putnam *et al.*, 2007 ▶).

Other *ab initio* approaches describe particles using an ensemble of scattering units that are optimized against experimental scattering data (Svergun, 1999 ▶; Chacón *et al.*, 1998 ▶; Glatter, 1980 ▶). Again, all models that yield the same *p*(*r*) function must be considered as equivalent. Furthermore, the approach requires that the maximum dimension, *D*_max_, of the particle is extracted from the scattering data, usually by an indirect Fourier transform (Svergun, 1992 ▶; Glatter, 1979 ▶). Even though several recommendations on good practice for the determination of *D*_max_ have been made (Jacques & Trewhella, 2010 ▶), small variations (∼10%) in its value in general yield the same quality parameters for the fit with the original SAS curve. The accuracy of retrieving geometric bodies from their back-calculated scattering curves by *ab initio* approaches has been discussed (Volkov & Svergun, 2003 ▶). In summary, the method is more efficient for globular than for anisometric particles and several models generated with the same setup should be depicted to illustrate variability and conserved features.

### 2.2. Rigid-body modelling   

In contrast to *ab initio* approaches, rigid-body modelling of complexes assumes that preliminary structural information on their constituent subunits is available. This may be high-resolution (crystallography or NMR) structures of the isolated subunits or appropriate homology models. Obviously, a critical point is the assumption that the structures from the isolated partners do not change significantly upon assembly into a complex. While small conformational changes may be tolerated (Gabel, 2012 ▶), larger ones will lead to erroneous interpretations. A well suited experimental method to assess the absence (or presence) of major conformational changes is by comparing NMR chemical shift perturbations (CSPs) from a free and a bound subunit (Williamson, 2013 ▶): if residues displaying CSPs are clustered within specific areas of the protein surface, it indicates that these surfaces are involved in interaction with other partners in the complex. However, if CSPs are distributed throughout the protein volume, major conformational changes might occur. It should be noted that the answers from CSPs are not always clear-cut and that some experience is required to interpret them.

Another important point to consider, in particular when combining high-resolution models from subunits obtained by crystallography, is the completeness of structures (*e.g.* in terms of amino-acid residues) with respect to the construct actually measured by SAS in solution. C-terminal, N-terminal or flexible internal loops are often not ‘seen’ by crystallography, but the presence of their scattering mass is necessary for an accurate interpretation of SAS data. Likewise, if His tags are present in the constructs measured in solution they should be incorporated in the modelling. Often, their mere presence at the right position of the structure is sufficient, in particular when their relative mass is small compared with the rest of the structure (Garcia-Saez *et al.*, 2011 ▶).

Several rigid-body modelling approaches use grids and incremental changes of the positions and orientations of the partners to refine a complex against SAS data (Petoukhov & Svergun, 2005 ▶; Konarev *et al.*, 2001 ▶). Usually, a single structure displaying the best χ^2^ fit is identified as the solution. This procedure, however, does not allow an appreciation of the stability and the uniqueness of the model obtained; *i.e.* do alternative solutions exist with a similar (or even lower) χ^2^ value and what are the residual (translational and rotational) degrees of freedom of the partners compatible with the SAS data? Rather than showing a single structure, an NMR-inspired approach of showing a family (ensemble) of models in agreement with all SAS restraints would allow the stability, uniqueness and residual degrees of freedom of refined models to be appreciated and visualized. To our knowledge, this important problem has not been solved so far in the general case (*i.e.* both rotational and translational degrees of freedom). However, a numerical solution has been proposed for the special case of a given fixed orientation of two subunits (Gabel *et al.*, 2006 ▶, 2008 ▶). In analogy to classical mechanics, the parallel axes theorem (Hoppe *et al.*, 1975 ▶; Engelman & Moore, 1975 ▶; Serdyuk & Fedorov, 1973 ▶) can be used to limit the conformational space by restricting the distance between two subunits around a specific, constant value in modelling approaches. Knowing their individual radii of gyration, *R*_1G_ and *R*_2G_, and having measured the overall *R*_G_ of the complex, the distance *r* between the bodies can be expressed as follows:
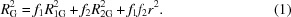
Here, 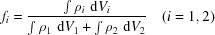
are the relative scattering ‘masses’ of the two particles with respective scattering-length densities ρ_*i*_ and volumes *V*_*i*_.

## 3. SAS–NMR rigid-body modelling   

NMR is presently arguably the most powerful and versatile technique to complement SAXS/SANS, with both techniques sharing similar experimental conditions (sample state, amount, concentration and labelling schemes). NMR complementary restraints include subunit orientations *via* residual dipolar couplings (RDCs), binding surfaces between subunits *via* chemical shift perturbations (CSPs) and distance restraints between pairs of residues *via* paramagnetic relaxation enhancements (PREs). A detailed discussion of NMR restraints and recent publications combining both techniques is beyond the scope of this review article and can be found elsewhere (Hennig & Sattler, 2014 ▶; Carlomagno, 2014 ▶; Madl *et al.*, 2011 ▶). Here, we illustrate, in the first part, the influence of progressively adding additional restraints to SAS data in the case of a model system composed of two non-identical, tri-axial ellipsoids. The results are analyzed and discussed in terms of the spatial distribution of models (with respect to a target structure) and in terms of χ^2^. In the second part, the state of the art of SAXS/SANS/NMR approaches is discussed in the light of the recent study of an archeal 390 kDa C/D box protein–RNA complex (Lapinaite *et al.*, 2013 ▶).

### 3.1. Uniqueness of structures as a function of SAS and complementary restraints: a model system consisting of two tri-axial ellipsoids   

**Figure 2:**
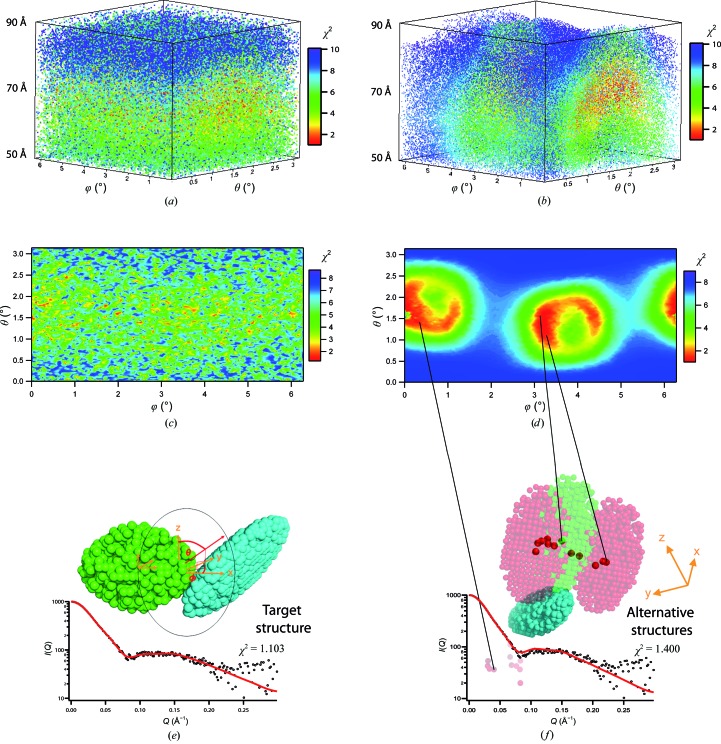
Colour-coded spatial distribution of conformations of a two-ellipsoid system illustrating the effect of progressive activation of SAS and NMR restraints. θ/ϕ designate the polar/azimuthal angles of the 40/30/20 Å (green) ellipsoid with respect to the common centre of mass [see (*e*)]. Warm colours (red, orange…) indicate good fits and cold colours (blue, violet) indicate poor fits against the reference SAXS curve calculated from the target model shown in (*e*). (*a*) Spatial distribution of the centre of the small (green) ellipsoid as a function of the polar angles (θ/ϕ) using arbitrary orientations of both ellipsoids and an inter-ellipsoid distance that varies between the target distance (70 Å) ±30%. (*b*) As in (*a*) but both ellipsoids are orientated as in the target structure. (*c*) Cross-section of (*a*) at the target distance (70 Å). (*d*) Cross-section of (*b*) at the target distance (70 Å). (*e*) Target model (θ/ϕ = 0.5π/π) and reference χ^2^ fit against its noise-endowed SAXS data. (*f*) Spatial distribution of 23 alternative structures (out of 2000 calculated) that are in excellent agreement (χ^2^ < 1.5) with the SAXS data of the target structure (transparent green). (The 50/20/10 Å cyan ellipsoid has been superposed for all structures.) The 23 structures are represented by red spheres indicating their centres of mass. Several of them correspond to symmetric solutions (transparent red spheres) which are located in a plane behind the cyan partner [red zone on the left-hand side of (*d*)]. For reasons of clarity, only two alternative models are depicted fully in transparent red colour. The fit against the reference SAXS data is from the left model. Please note the rotated *xyz* reference frame and model orientation with respect to (*e*) which was applied here for reasons of clarity.

We illustrate the effect of structural restraints by a pedagogical example of a model system consisting of two non-identical, tri-axial ellipsoids (Fig. 2 ▶ *e*). The two ellipsoids were generated using *DAMMIN* (Svergun, 1999 ▶) with half-axes of 40/30/20 Å and 50/20/10 Å containing 873 and 1134 dummy beads, respectively. A SAXS curve was calculated from the initial ‘target’ configuration (centre-to-centre distance 70 Å) using *CRYSOL* (Svergun *et al.*, 1995 ▶) in the default setup and endowed with noise comparable to a 1 s SAXS data frame from BSA (concentration ∼5 mg ml^−1^) as measured on the ESRF BioSAXS beamline BM29. Using these simulated SAXS data as a reference ‘target’ structure (Fig. 2 ▶ *e*), the agreement of alternative conformations (the positions and/or orientations of both ellipsoids) were scored in a least χ^2^ fit following a recently developed protocol (Gabel, 2012 ▶). The results are plotted as a function of the polar and azimuthal angles θ/ϕ which define the position of the centre of mass of the 40/30/20 Å (green) ellipsoid with respect to the common centre of mass (Fig. 2 ▶ *e*). Selected structural restraints were progressively activated as follows.

(i) No restraints: arbitrary orientations of both ellipsoids at a centre-to-centre distance corresponding to the target structure distance ± 30% (100 000 structures; Fig. 2 ▶ *a*).
(ii) Elementary restraints from SAS (parallel axes theorem): arbitrary orientations but the distance between both centres is fixed and identical to that of the target structure (10 000 structures, Fig. 2 ▶ *c*).
(iii) Only NMR restraints in the form of residual dipolar couplings (RDCs): respective orientations fixed and identical to those of the target structure but with the centre-to-centre distance varied by ±30% (100 000 structures, Fig. 2 ▶ *b*).
(iv) SAS distance restraints and NMR RDCs: both orientations and centre-to-centre distance identical to those of the target structure (10 000 structures, Fig. 2 ▶ *d*).

As illustrated in Fig. 2 ▶(*a*), there is no pronounced clustering of good models in the absence of structural restraints (arbitrary ellipsoidal orientations and positions). Good models are found around and below the target distance and very large distances are severely penalized. Even at the correct target distance (70 Å), good solutions are distributed more or less randomly in the θ/ϕ plane (Fig. 2 ▶ *c*). Once the ellipsoids are in the correct target orientation, a specific pattern/clustering of good solutions emerges (Fig. 2 ▶ *b*). When, in addition, only models with the target distance are considered (Fig. 2 ▶ *d*), the ensemble of good models showing low χ^2^ values (Fig. 2 ▶ *f*) is confined to restricted regions in the conformational space as predicted by theory (Gabel *et al.*, 2006 ▶). In conclusion, these results illustrate that even in a very favourable situation (known high-resolution models, orientations and distance between partners) a single structural solution cannot necessarily be identified. In general, an ensemble (or family) of structures (red areas in Fig. 2 ▶ *d* and models in Fig. 2 ▶ ▶ *f*) emerges whose spatial distribution will depend on the geometry of the partners and the *Q*-range and noise level of the SAS data available.

### 3.2. State of the art of NMR–SAS approaches   

The previous section illustrates impressively that in rigid-body modelling using only SAS (or SAS and NMR restraints) the conformational space of ‘good’ structures can be rather extended, *i.e.* several structures can have a similar χ^2^ to the target structure itself. Adopting an ‘NMR-inspired’ approach (*i.e.* showing a family of structures in agreement with all structural restraints) is therefore more informative than showing only a single model as ‘the’ solution and visualizes the variability and spatial distribution of structural solutions that are in agreement with all data available.

A recent example of the state of the art in hybrid NMR–SAXS/SANS approaches has been the determination the apo and holo structures of the 390 kDa box C/D complex (Lapinaite *et al.*, 2013 ▶): the structure of this complex, which methylates ribosomal RNA, has been obtained by a combination of NMR chemical shift perturbations (CSPs), paramagnetic relaxation enhancements (PREs) and distance restraints from both SAXS and SANS. Using available high-resolution (crystallo­graphy) building blocks of the protein and RNA compounds, the authors have determined subunit contacts with CSPs and distances and orientations by PREs. While SAXS was used to validate the overall shape, the positions of the subunits within the complex were confined using deuterium labelling in combination with SANS and contrast variation, an approach that was particularly helpful to describe the respective protein positions as well as the RNA shape within the complex. Adhering to NMR philosophy, an ensemble of structures satisfying all structural restraints (along with a model displaying the lowest χ^2^) was presented. This example shows in an impressive way that even large biomacromolecular complexes are nowadays accessible to hybrid NMR–SAS approaches when realistic building blocks are available. However, it also illustrated that for such complexes SAXS should be complemented by SANS to reveal the internal quaternary structure accurately.

## 4. Influence of a hydration shell   

SAXS and SANS are sensitive, respectively, to electronic and nuclear scattering-length density fluctuations on a length scale of the order of nanometres (Trewhella, 1990 ▶). These fluctuations usually occur between the solutes (biomacromolecules) and the solvent (in most cases an aqueous buffer). While the bulk solvent is generally considered to be homogeneous on SAS length scales, it has been found that the solvent in the immediate vicinity (approximately a few Å) of the solute molecules can have a different average density to the bulk solvent and is usually slightly denser (Merzel & Smith, 2002*b* ▶; Svergun *et al.*, 1998 ▶). This region, known as the hydration shell, has been implemented in a number of programs to back-calculate SAXS (and, more rarely, SANS) curves from atomic structures, either as a shell of a specific thickness (Sinibaldi *et al.*, 2008 ▶; Svergun *et al.*, 1995 ▶, 1998 ▶), as grid elements (Bardhan *et al.*, 2009 ▶; Tjioe & Heller, 2007 ▶), as dummy atoms (Schneidman-Duhovny *et al.*, 2010 ▶), as explicit water molecules (Grishaev *et al.*, 2010 ▶; Yang *et al.*, 2009 ▶; Park *et al.*, 2009 ▶; Merzel & Smith, 2002*a* ▶), as a density map (Poitevin *et al.*, 2011 ▶; Virtanen *et al.*, 2010 ▶) or by voxelization (Liu *et al.*, 2012 ▶). Here, we limit our discussion to the influence of a hydration shell on rigid-body modelling by considering two simplified pedagogical examples: (i) variations of the SAXS and SANS curves of a spherical molecule as a function of its geometric radius and the density of the hydration shell and (ii) the accuracy of the distance between two spherical molecules as a function of their hydration shells. Both examples have the advantage that they can be expressed analytically. The findings can be transferred in a qualitative manner to more complex and realistic cases.

### 4.1. Spherical molecule with a homogeneous hydration shell   

**Figure 3:**
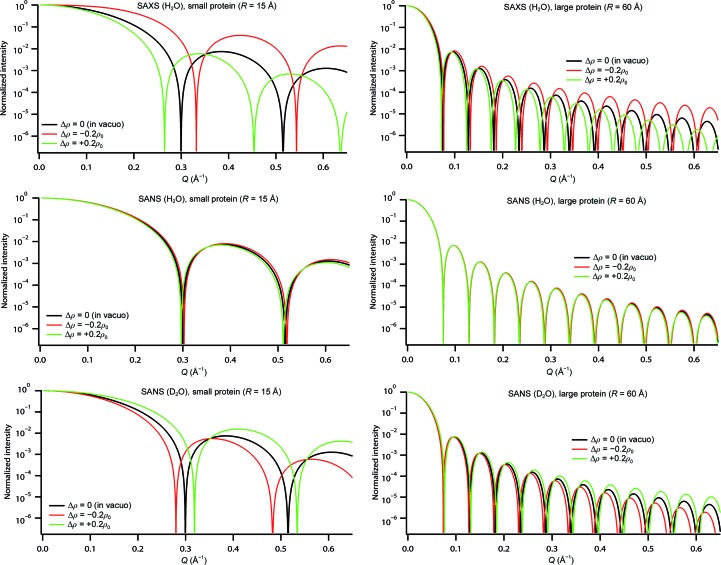
SAXS and SANS (H_2_O and D_2_O) curves at different contrast conditions for a small and a large spherical molecule including a homogeneous hydration shell of 3 Å thickness of three different densities.

The intensity scattered by a spherical molecule with radius *R* and a homogeneous hydration shell of thickness *d* can be derived from the original equation of a sphere by Rayleigh by including an additional concentric shell (Pedersen, 2002 ▶),
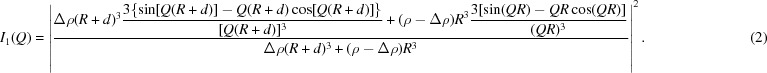
ρ and Δρ are the scattering-length density differences of the molecule and the hydration shell with the bulk solvent, respectively. As a function of contrast and radiation, they can be either positive or negative. Fig. 3 ▶ and Table 2 ▶ provide an overview of scattering curves and radii of gyration, *R*_G_, calculated for (hydrogenated) proteins of geometrical radii *R* = 15 and 60 Å measured by SANS in H_2_O and D_2_O and by SAXS in H_2_O. In the calculations the hydration shell was assumed to have a thickness *d* = 3 Å with Δρ = −0.2ρ_0_, 0 (protein *in vacuo*) and +0.2ρ_0_ (ρ_0_ being the scattering-length density of the bulk solvent). ρ was chosen as 2.4 and −4 × 10^10^ cm^−2^ for SANS in H_2_O and D_2_O, respectively, and the corresponding ρ_0_ as −0.56 and 6.4 × 10^10^ cm^−2^ (Jacrot, 1976 ▶). For SAXS, ρ was chosen as 0.44 and ρ_0_ as 0.33 e^−^ Å^−3^ (Putnam *et al.*, 2007 ▶).

Both the scattering curves and the *R*_G_ vary significantly in the presence of a hydration shell. The effects are most pronounced for small proteins: a density variation of ±20% of the hydration shell can lead to *R*_G_ variations of 15% or more (SAXS and SANS in D_2_O). Variations of this order have been measured experimentally (Svergun *et al.*, 1998 ▶). In addition, the positions of the maxima and minima of the scattering curves are displaced by up to 10% and the relative heights of the maxima vary as well. It should be noted that the relative change in the *R*_G_ and the positions of the maxima and minima depends on the radiation and contrast and can be of opposite sign for the same particle in the same buffer. SAXS/SANS in combination with contrast variation therefore represents a powerful tool to characterize the structural properties of the hydration shell. Importantly, the effect of a hydration shell is very small for SANS in H_2_O. This is owing to the very small neutron scattering-length density of water. It is zero for ∼8%D_2_O/92%H_2_O (Jacrot, 1976 ▶), which is equivalent to measuring biomacromolecules *in vacuo*. This situation is unique to neutrons and cannot be obtained with X-rays.

The impact of a hydration shell on the scattering curve diminishes as the particle size increases. For spherical proteins with a diameter of ∼120 Å the relative changes in the *R*_G_ are about 3–4% under the most sensitive experimental conditions (SAXS; Table 2 ▶) and significant changes of the positions of the minima and maxima are only visible at high angles where the experimental signal is low and the noise level is elevated.

In conclusion, it is imperative to take the effects of a hydration shell into account for model building and refinement of small biomacromolecules from SAXS data and from SANS data in D_2_O. The effects can be neglected in most cases for SANS in H_2_O. While the results above have been obtained in the special case of spherical particles, more general equations exist for other simple geometric bodies such as ellipsoids of revolution and tri-axial ellipsoids (Pedersen, 2002 ▶) and can be generalized to include a concentric hydration shell. However, they cannot be solved analytically. Calculations for atomic structures can be made by using some of the existing programs: in general, the larger the relative volume of the hydration shell with respect to the particle volume and the larger its thickness with respect to the particle dimensions, the more important its relative contribution. It is therefore particularly important for the modelling of small, but also for elongated and unfolded (disordered), proteins.

### 4.2. Influence of a hydration shell on the refinement of a two-body system   

**Figure 4:**
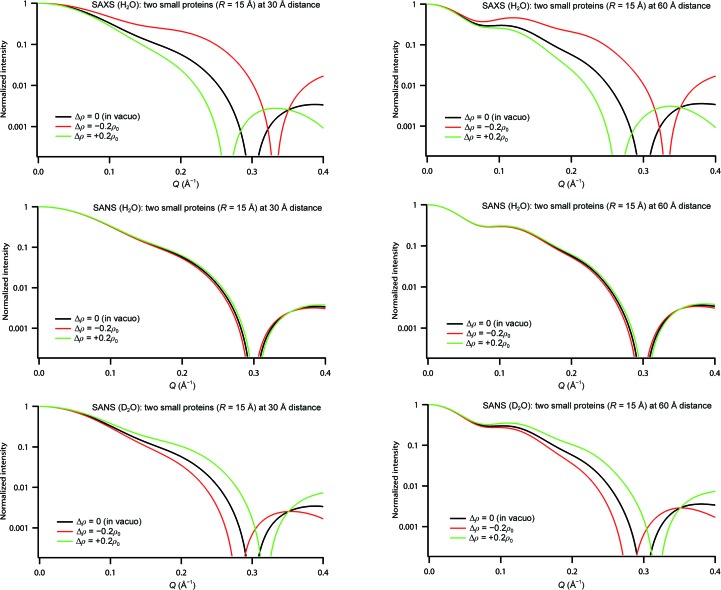
SAXS and SANS (H_2_O and D_2_O) curves of two spheres of identical radii (15 Å), separated by 30 Å (left) and 60 Å (right), including identical homogeneous hydration shells (*d* = 3 Å) of variable density.

The findings for a single sphere can be extended to a two-body system (*e.g.* a two-domain protein, protein–protein or protein–RNA/DNA complex). Again, we consider a simplified system consisting of two spheres in order to illustrate the effects qualitatively. We focus here on the accuracy of the inter-particle distance determined from SAS data. The scattered intensity from a system composed of several spheres was originally determined by Debye and can be calculated for the specific case of two identical spheres of geometric radius *R* at a distance (centre to centre) *r* by including hydration shells as in the previous section (Pedersen, 2002 ▶):
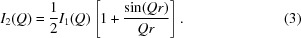

*I*_1_(*Q*) is the expression of a single sphere of radius *R* and with a hydration shell of thickness *d* (2). Fig. 4 ▶ and Table 3 ▶ show the calculated SAXS and SANS curves as well as the *R*_G_ for two identical spheres (*R* = 15 Å), including hydration shells, at two specific distances (30 and 60 Å). As in the case of a single sphere, the scattering curves and *R*_G_ depend strongly on the presence of a hydration shell for SAXS and for SANS in D_2_O, in particular for the system with a compact geometry (*r* = 30 Å). The sign of the relative change in *R*_G_ and the direction of the shift of the maxima and minima with respect to the *in vacuo* structures, as a function of radiation and solvent conditions, are similar to the case of a single sphere. Again, SANS in H_2_O is the experimental condition least sensitive to the presence of a hydration shell.

The distance between two partners is determined by the parallel axes theorem (1). When the hydration shell is taken into account, the integrals run over the atomic volumes and the hydration shells. For two identical spheres one has *f*_1_ = *f*_2_ = 1/2 and *R*_1G_ = *R*_2G_ and the inter-sphere distance is *r* = 2(*R* ^2^_G_ − *R* ^2^_1G_). What is the error of *r* in a modelling process when using the parallel axes theorem with an experimentally measured *R*_G_ of the complex but the radii of gyration of the individual partners from their atomic coordinates *in vacuo* (*i.e.* ignoring their hydration shells)? Table 3 ▶ shows that under unfavourable conditions the error can be as elevated as 10%. It is therefore imperative to take the presence of a hydration shell into account for rigid-body modelling of a two (or more) body system.

## 5. Conclusions and perspectives   

In the present contribution we have provided a short overview of the general aspects of the uniqueness of models derived from SAS data and focused in more detail, using pedagogical examples, on the effects of incorporating NMR restraints (in particular subunit orientations) and the influence of a hydration shell on the uniqueness and variability of structural models.

We found that the incorporation of a hydration shell is essential for accurate modelling when smaller biomacro­molecules (*e.g.* ubiquitin or lysozyme) are measured by SAXS (in H_2_O or D_2_O) or by SANS in D_2_O. The important families of intrinsically disordered proteins (IDPs), linker-connected multi-domain proteins and RNA/DNA-binding proteins as well as small structural RNAs are especially considered. The main parameters affected by neglecting the contribution of a hydration shell in these cases will be an over/underestimation of the dimensions of individual particles or of the distance between two partners in a modelling process, *e.g.* if the parallel axes theorem is applied. Several programs (reviewed in §[Sec sec4]4) have been developed to take the hydration shell into account. An interesting question related to the hydration shell is how it is correlated to the surface charge (distribution) of particles in solution, which is particularly important in the case of charged proteins and RNA/DNA molecules. As illustrated in Fig. 3 ▶, combined SAXS/SANS studies of appropriate systems are very efficient to provide useful insight into this interesting topic.

NMR restraints in solution are particularly powerful in hybrid SAS approaches owing to their complementarities (§[Sec sec3]3). Presently, NMR–SAS hybrid approaches are being actively developed to address challenging structural questions from complex biomacromolecular systems (Hennig & Sattler, 2014 ▶; Carlomagno, 2014 ▶; Madl *et al.*, 2011 ▶). When atomic models of multi-subunit complexes are determined by SAS–NMR approaches based on building blocks, it is particularly important to assess their uniqueness and variability. Fig. 2 ▶ provides a visual representation of the accuracy that can be obtained by such approaches by illustrating the spatial distribution of models in agreement with the available data. We believe that this kind of representation, which has been adopted by the NMR community (*i.e.* showing a family of models rather than a single model), should be applied generally when showing models of multi-subunit systems using SAS (and NMR) data. However, only numerical solutions have been found so far for the specific case of subunits of known orientation (Gabel *et al.*, 2006 ▶), and further mathematical developments are needed to predict the conformational space analytically in the general case of arbitrary orientations.

Fig. 2 ▶(*d*) illustrates that additional distance restraints are very useful to reduce the conformational space of good solution further and should therefore be applied when available. These include pairwise distance restraints between specific residues provided by NMR PREs (Hennig & Sattler, 2014 ▶; Carlomagno, 2014 ▶; Madl *et al.*, 2011 ▶) and also recent developments such as heavy-atom labelling in SAS (Grishaev *et al.*, 2012 ▶) or multi-wavelength anomalous X-ray scattering of specific chemical groups (Makowski *et al.*, 2012 ▶). The feasibility of the latter approach has also been demonstrated for neutrons using single atoms as labels (Seeger *et al.*, 1997 ▶) but is more limited by signal to noise and available labels. The development of new deuterium-labelling schemes of specific chemical groups or parts of proteins, in particular segmental labelling (Hennig & Sattler, 2014 ▶), is another promising approach for SANS in the near future.

Finally, several exciting recent approaches such as online HPLC systems (Round *et al.*, 2013 ▶; Berthaud *et al.*, 2012 ▶), cryo-SAXS (Meisburger *et al.*, 2013 ▶) and X-ray free-electron lasers (Pérez & Nishino, 2012 ▶) demonstrate that SAS is very dynamic at the moment and further valuable contributions in structural biology can be expected in the near future.

## Figures and Tables

**Table 1:** A list of recent examples where SAS was used in combination with various other biophysical techniques to improve the quality and accuracy of the structural models being generated

(Major) complementary technique	Principal findings/contributions	References
X-ray crystallography	High-resolution structural models	Elegheert *et al.* (2012 ▶), Ando *et al.* (2011 ▶), Clerici *et al.* (2009 ▶)
	Subunit atomic building blocks for SAS or EM	
	Conformation *in cristallo versus* in solution	
EM	EMSAS combinations improve the reliability of models of unknown high-resolution structure	Breyton *et al.* (2013 ▶), Neves *et al.* (2012 ▶), Jensen *et al.* (2011 ▶)
	EM generally yields higher nominal resolution than SAS	
	SAS is more sensitive to multiple conformations and/or flexible parts	
MS	Identification of oligomeric states	Wang *et al.* (2011 ▶)
	Refinement of SAS models	
AUC/SPR/FRET/ITC	Specific binding stoichiometry	Appolaire *et al.* (2013 ▶), Vijayakrishnan *et al.* (2011 ▶), Ghachi *et al.* (2011 ▶) (AUC), Mertens *et al.* (2012 ▶), Kulczyk *et al.* (2012 ▶), Kim *et al.* (2011 ▶) (SPR), Street *et al.* (2011 ▶) (FRET), Hilge *et al.* (2009 ▶), Jenkins *et al.* (2008 ▶) (ITC)
	Oligomerization properties	
	Thermodynamics	
	Kinetics and affinity	
	Subunit content and architecture	

**Table 2:** Radii of gyration of spherical molecules including a homogeneous hydration shell of different density to the bulk solvent

Particle		*R*_G_ ()
SAXS small (H_2_O)	0.2	13.2
	0.0	11.5
	0.2	4.9
SAXS large (H_2_O)	0.2	47.7
	0.0	46.2
	0.2	44.3
SANS small (H_2_O)	0.2	11.7
	0.0	11.5
	0.2	11.3
SANS large (H_2_O)	0.2	46.3
	0.0	46.2
	0.2	46.1
SANS small (D_2_O)	0.2	9.6
	0.0	11.5
	0.2	12.6
SANS large (D_2_O)	0.2	45.3
	0.0	46.2
	0.2	47.0

**Table 3:** *R*_G_ and distances for a two-sphere system as a function of radiation *R*_G_ values were extracted with a Guinier fit (Guinier, 1939 ▶) from the data and the inter-sphere distance *r* was calculated with the parallel axes theorem (1) from the spheres without hydration shells.

Sample		*R*_G_ ()	*r*, calculated ()	*r*, real ()
SAXS (H_2_O)	0.2	19.9	32.4	30.0
	0.0	18.8	29.8	
	0.2	15.6	21.0	
SAXS (H_2_O)	0.2	32.8	61.4	60.0
	0.0	32.1	60.0	
	0.2	30.3	56.0	
SANS (H_2_O)	0.2	18.7	29.4	30.0
	0.0	18.8	29.8	
	0.2	18.9	30.0	
SANS (H_2_O)	0.2	32.1	60.0	60.0
	0.0	32.1	60.0	
	0.2	32.2	60.2	
SANS (D_2_O)	0.2	17.7	27.0	30.0
	0.0	18.8	29.8	
	0.2	19.5	31.4	
SANS (D_2_O)	0.2	31.5	58.6	60.0
	0.0	32.1	60.0	
	0.2	32.5	60.8	
